# Comparison of Standard Clinical and Instrumented Physical Performance Tests in Discriminating Functional Status of High-Functioning People Aged 61–70 Years Old

**DOI:** 10.3390/s19030449

**Published:** 2019-01-22

**Authors:** Alice Coni, Jeanine M. Van Ancum, Ronny Bergquist, A. Stefanie Mikolaizak, Sabato Mellone, Lorenzo Chiari, Andrea B. Maier, Mirjam Pijnappels

**Affiliations:** 1 Department of Electrical, Electronic and Information Engineering “Guglielmo Marconi” (DEI), University of Bologna, 40136 Bologna, Italy; alice.coni2@unibo.it (A.C.); sabato.mellone@unibo.it (S.M.); lorenzo.chiari@unibo.it (L.C.); 2 Department of Human Movement Sciences, @AgeAmsterdam, Faculty of Behavioural and Movement Sciences, Vrije Universiteit Amsterdam, Amsterdam Movement Sciences, 1081 BT Amsterdam, The Netherlands; j.m.van.ancum@vu.nl (J.M.V.A.); Andrea.Maier@mh.org.au (A.B.M.); 3 Department of Neuromedicine and Movement Science, Norwegian University of Science and Technology, 7491 Trondheim, Norway; ronny.bergquist@ntnu.no; 4 Department of Clinical Gerontology, Robert Bosch Medical Foundation, 70376 Stuttgart, Germany; Stefanie.Mikolaizak@rbk.de; 5 Health Sciences and Technologies—Interdepartmental Center for Industrial Research (HST-ICIR), University of Bologna, 40126 Bologna, Italy; 6 Department of Medicine and Aged Care, @AgeMelbourne, University of Melbourne, Royal Melbourne Hospital, Melbourne, VIC 3050, Australia

**Keywords:** instrumented assessments, smartphone, standard clinical measures, physical function

## Abstract

Assessment of physical performance by standard clinical tests such as the 30-s Chair Stand (30CST) and the Timed Up and Go (TUG) may allow early detection of functional decline, even in high-functioning populations, and facilitate preventive interventions. Inertial sensors are emerging to obtain instrumented measures that can provide subtle details regarding the quality of the movement while performing such tests. We compared standard clinical with instrumented measures of physical performance in their ability to distinguish between high and very high functional status, stratified by the Late-Life Function and Disability Instrument (LLFDI). We assessed 160 participants from the PreventIT study (66.3 ± 2.4 years, 87 females, median LLFDI 72.31, range: 44.33–100) performing the 30CST and TUG while a smartphone was attached to their lower back. The number of 30CST repetitions and the stopwatch-based TUG duration were recorded. Instrumented features were computed from the smartphone embedded inertial sensors. Four logistic regression models were fitted and the Areas Under the Receiver Operating Curve (AUC) were calculated and compared using the DeLong test. Standard clinical and instrumented measures of 30CST both showed equal moderate discriminative ability of 0.68 (95%CI 0.60–0.76), *p* = 0.97. Similarly, for TUG: AUC was 0.68 (95%CI 0.60–0.77) and 0.65 (95%CI 0.56–0.73), respectively, *p* = 0.26. In conclusion, both clinical and instrumented measures, recorded through a smartphone, can discriminate early functional decline in healthy adults aged 61–70 years.

## 1. Introduction

Early identification of people at risk of functional decline is essential for targeting preventive interventions for the ones at risk. Physical function is one’s ability to carry out discrete actions or activities of daily living [1] and can be reliably assessed with questionnaires such as the Late-Life Function and Disability Instrument (LLFDI) [2,3]. Although the application of these instruments is recommended and clinically useful to identify people at risk or assess changes over time, they have some limitations. For instance, they may suffer from floor or ceiling effects, and since they are self-reports, the accuracy of the data collected could be affected by social desirability or response biases [4].

Physical performance is one domain of physical function that can be objectively measured using standard clinical tests, such as counting repetitions in the 30-s Chair Stand Test (30CST) and timing duration of a Timed up and Go test (TUG) [5,6,7]. Although the standard clinical outcomes of these physical performance tests are commonly used assessing older or patient populations [5,6], their ability to detect early signs of functional decline in relatively healthy and fit older adults is not clear.

Instrumented assessments with the use of inertial sensors allow objective measurements of the quality of the task and its (sub-)movements while performing such physical performance tests [8]. Recent studies demonstrated that features obtained with inertial sensors, alone or in combination with the standard clinical outcome, can be of added value for identification or prediction of physical function, without compromising the simplicity of testing [9,10]. Furthermore, it was shown that instrumented physical performance tests were more strongly related to health status, functional status, and daily physical activity compared to the manually recorded version of the tests [11]. Still, the potential ability of such features to detect slight changes in functional status for an early detection of functional decline, when preventive and/or protective actions can be put in place, needs further investigation.

The aim of this study was to assess whether standard clinical measures of physical performance and instrumented measures collected through a smartphone during 30CST and TUG tests, can distinguish between older individuals with a High and Very High Functional Status, stratified by the LLFDI.

## 2. Materials and Methods

### 2.1. Population

To investigate the potential of standard clinical and instrumented measures in discriminating at high functional status, data from the baseline cohort of the H2020 PreventIT project [12] were analyzed. PreventIT [13] is a three-armed multicenter trial with three centers in Trondheim (Norway), Amsterdam (The Netherlands), and Stuttgart (Germany). The treatment arms include two behavior change exercise programs and a control group. It makes use of a new ICT-based behavioral change approach for young older adults for preventing functional decline and for motivating people to take care of their own health. Participants were invited by a random draw from local registries and included if they were (i) aged between 61 and 70 years, (ii) retired for more than six months, (iii) home-dwelling, (iv) able to read newspaper or text on smartphone (SP), (v) able to walk 500 m without walking aids, (vi) without cognitive impairments (Montreal Cognitive Assessment, MoCA > 24 points [14]), and (vii) they were excluded if they participated in exercise classes more than once a week or did sport for more than 150 min per week.

Within the larger PreventIT cohort, 160 participants (mean age 66.3 ± 2.4 years, 87 females) who met the inclusion/exclusion criteria also performed both the instrumented 30CST and TUG tests. During the baseline assessment, participants filled questionnaires about age, gender, body mass index (BMI), physical activity (PA), hand grip strength (HAND [15]), and cognitive status (MoCA [14]).

### 2.2. Outcome

The Late-Life Function and Disability Instrument (LLFDI) was used to measure the functional status of participants [16]. The LLFDI evaluates both function and disability, assessing the poor ability to perform specific physical tasks encountered in daily routines. The function component, which was used in this study, evaluates self-reported difficulty to perform 32 activities in daily living consisting of three dimensions: upper extremity, basic lower extremity, and advanced lower extremity. Questions are phrased, “How much difficulty do you have doing a particular activity without the help of someone else and without the use of assistive devices?” with a rating scale from 1 to 5 (the higher the scoring category, the less difficulty the person has in doing activities). The overall function raw score is obtained adding the scores of all the 32 items [2].

As no validated cut-off has been described in literature to distinguish between people with different levels of functional status, we dichotomized the scaled scores (ranged 0 to 100) of the function domain of the LLFDI based on the median value to classify the people in our cohort as high (HFS) and very high (VHFS) functional status.

### 2.3. Standard Clinical Physical Performance Tests

The physical performance of participants was objectively assessed by two physical performance tests under standard instructions given by the assessors: the 30CST and the TUG. During the 30CST, participants started seated, on the command “go”, they stood up and sat down repeatedly for 30 s as quickly as they could. The total number of repetitions performed during the 30CST were counted by the assessors as standard clinical outcome of the 30CST. During the TUG, participants started seated on a chair, on the command “go”, they rose from the chair, walked three meters ahead at a comfortable and safe pace, made a 180° turn, walked back to the chair, and sat down again. The stopwatch-based total time needed to perform the TUG test was recorded by assessors as standard clinical outcome of the TUG.

### 2.4. Instrumented Physical Performance Tests

While performing the two physical performance tests, participants were instrumented with a smartphone on their lower back (at the level of the 5th lumbar spine) through a waist-worn elastic belt. The smartphone-based system was developed within the FARSEEING project [17]. A custom Android application [18] running on the smartphone (Galaxy SIII, Samsung, sampling frequency 100 Hz, accelerometer ± 2 g, gyroscope ± 250 °/s) was used for recording the Triaxial components of inertial signals: Antero-Posterior (AP), Medio-Lateral (ML), and Vertical (V). The instrumented features computed from the collected inertial signals were used as instrumented outcome of the physical performance tests. Triaxial inertial signals were processed using MATLAB [19] to extract a set of instrumented features [20]. 

**Figure 1:**
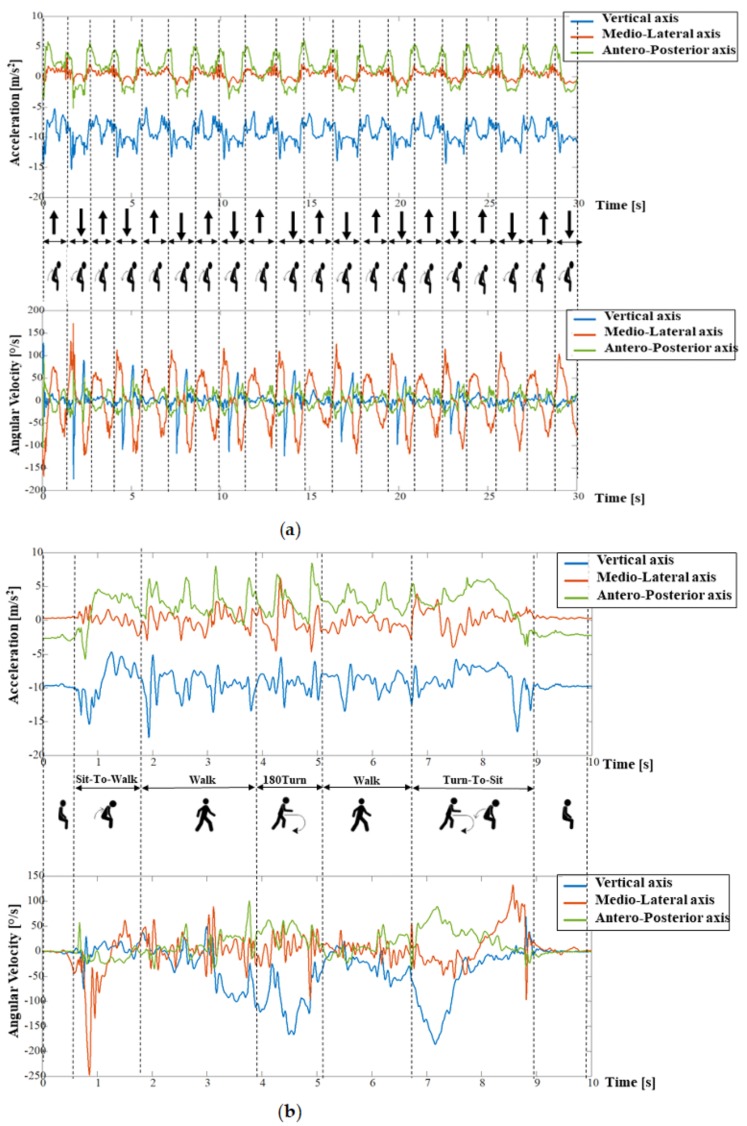
Time series of acceleration and angular velocity of the two instrumented physical performance tests. (**a**) Time series of acceleration and angular velocity over the Sit-to-Stand (↑) and Stand-to-Sit (↓) subphases of the 30CST and (**b**) time series of acceleration and angular velocity over the subphases of the TUG cycles.

Signals recorded during the 30CST were first segmented into two subphases: Sit-to-Stand and Stand-to-Sit transitions (Figure 1a). The AP acceleration signal and the angular velocity about the ML axis were used to identify postural transitions [21]. Twenty-one instrumented features were extracted from the 30CST test [21,22,23], including durations, measures of the intensity (Root Mean Square, RMS, m/s^2^) and smoothness (Normalized Jerk Score, NJS, m) in AP, ML, and V direction of each repetition. The features were computed for each Stand-to-Sit/Sit-to-Stand transition and then averaged over the Sit-to-Stand/Stand-to-Sit subphases (see Table 1).

The TUG was divided into four subphases: Sit-to-Walk, Walk, 180Turn, and Turn-to-Sit (Figure 1b). The AP acceleration and the angular velocity on the ML axis were used to identify postural transitions and the walking phase, and the angular velocity around the V axis was used to identify turns [21]. Walking features were derived from the AP, ML, and V signals, excluding postural transitions and the turning phase, and concatenating the two episodes of straight walk [24]. Twenty-eight features were extracted from the TUG test [21,22,23,25,26,27,28] including durations, intensity (RMS), and smoothness (NJS) of each subphase, as well as the mean and maximum angular velocity during the turns and the number of steps performed while walking and turning (see Table 2).

### 2.5. Statistical Analysis

Statistical analyses were performed in R for Windows version 3.4.3 [29]. Four logistic regression models were fitted and the area under the ROC Curve were compared to assess the performances of 30CST and TUG standard clinical and instrumented outcome measures in distinguishing between HFS and VHFS.

For each physical performance test, first a univariable logistic regression was fitted with the standard clinical measure as input (number of repetitions counted by assessor for 30CST and stopwatch-based total time in s for TUG). Secondly, a step-wise backward multivariable logistic regression with the instrumented features as input was fitted. Note that for the comparison between models, we excluded the standard measures (number of repetitions for 30CST or total duration for TUG, obtained with inertial sensors) from the analyses for the instrumented models, as this allowed evaluation of the discriminative ability of purely the more detailed features. To do so, the instrumented features were pre-processed with the same procedure for both 30CST and TUG. The jerk scores (NJS for all the subphases in AP, ML, and V direction), which were not normally distributed, were log-transformed and all the instrumented features were normalized to compare measures by z-scores. The linearity of each instrumented measure was assessed by fitting a restricted cubic spline function (using the R package “Hmisc” [30] with three knots at 0.1, 0.5, and 0.9 quantiles) in the logistic regression model. Usually, in order to avoid overfitting, the assessment of multicollinearity is recommended before fitting the multivariable logistic regression on the dataset. Furthermore, the validity of the multivariable logistic regression model becomes problematic when the ratio of the numbers of subjects per variable inserted in the model is less than 10 [31]. We addressed these issues by following the next steps. Firstly, the multicollinearity between instrumented features was assessed (R package “mctest” [32]). To detect and deal with multicollinearity (i) the Variance Inflation Factor (VIF) was computed on the entire dataset; (ii) the instrumented measure with highest VIF was selected and removed from the dataset; and (iii) the VIF was computed on the new subset of measures. The procedure was repeated until no collinearity was found (i.e., all the elements in the VIF vector were below 10). Starting from the obtained subset of instrumented feature, we selected those features that better discriminate between participants with HFS and VHFS (*p* ≤ 0.15) fitting one univariable logistic regression for each instrumented feature. The resulting subset of instrumented features was entered into a step-wise backward multivariable logistic regression. The features with *p* ≤ 0.05 were selected to fit the final model. 

To compare the standard clinical and instrumented models for both physical performance tests, the discriminative ability of the resulting models was assessed by comparing the Area Under the Receiver Operating Curve (AUC). We used the DeLong test to assess differences between AUC of the models [33] (*p* ≤ 0.05 was considered statistically significant). A bootstrapping method with backward step-down variable deletion (R package “rms” [34]) was applied to internally validate each model and assess the impact of outliers.

Finally, to compare the added value of the instrumented features to the standard clinical measures, a sensitivity analysis was conducted for both the 30CST and TUG tests on the discriminative ability in distinguishing between HFS and VHFS of the following three models: (i) standard clinical model, obtained from the standard clinical measure (30CST number of repetitions or TUG duration); (ii) instrumented model, obtained from the selected subset of instrumented features; and (iii) combined model, obtained by including the instrumented 30CST number of repetitions or TUG duration in the instrumented model.

## 3. Results

The baseline cohort consisted of n = 160 (age 66.3 ± 2.4 years, 87 females) strong and active (HAND 33.41 ± 11.19 kg, 90% declared a PA level ≥ 3) participants. The population was divided into two groups, based on the median value of the LLFDI score: HFS (LLFDI range: 44.33–71.33) and VHFS (LLFDI range: 72–100). Demographics of the total population and of both groups are reported in Table 3.

### 3.1. Standard Clinical Physical Performance Measures

The number of repetitions for the 30CST was higher in the VHFS than in the HFS (Table 3), with the discriminative ability, expressed as odds ratio (OR), determined by the univariable logistic regression of OR = 1.29 (95%CI [1.15–1.46]), *p* < 0.001).

For the TUG, the VHFS were faster than the HFS (Table 3), with a discriminative ability of OR 0.58, 95%CI [0.43–0.75] and *p* < 0.001).

### 3.2. Instrumented Physical Performance Measures

For the instrumented 30CST, six of the 21 features were excluded from the original datasets to avoid multicollinearity (Supplementary Table S1), resulting in 15 features for further analysis. From the univariable logistic regression, four features were selected (*p* ≤ 0.15) (Supplementary Table S2). Step-wise backward multivariable logistic regression analysis resulted in a model with three features with significant discriminative ability: “mean Stand-to-Sit G RMS MLs” (OR = 0.71, 95%CI [0.49 0.98], *p* = 0.045), “mean Duration Sit-to-Stand” (OR = 0.69, 95%CI [0.48 0.98], *p* = 0.041), and “SD Duration Sit-to-Stand” (OR = 0.62, 95%CI [0.41 0.89], *p* = 0.014).

For the instrumented TUG, four of the 29 features were excluded from the original datasets to avoid multicollinearity (Supplementary Table S3), resulting in 25 features for further analysis. From the univariable logistic regression analyses, nine features were selected (*p* ≤ 0.15) (Supplementary Table S4). Step-wise backward multivariable logistic regression analysis resulted in a model with two features with significant discriminative ability: “Walk duration” (OR = 0.59, 95%CI [0.38–0.86], *p* = 0.045) and “Turn-to-Sit Turning maximum velocity” (OR = 1.50, 95%CI [1.05–2.18], *p* = 0.031).

### 3.3. Comparison of AUC of Models with Standard Clinical, Instrumented, and Combined Measures

**Figure 2:**
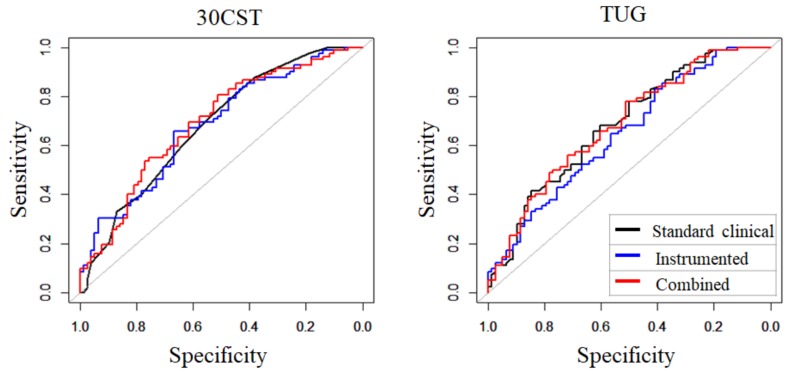
Discriminative ability (AUC and DeLong test) of standard clinical (black line), instrumented (blue line), and combined (red line) measures of the 30CST and TUG test.

Discriminative ability (AUC values) of each model is presented in Figure 2 and Table 4. The internal validation of each of the models was assessed by applying a bootstrapping method with backward step-down variable deletion (Supplementary Table S5). The original AUC and optimism-corrected AUCs were in the same range (with differences less than 0.04), indicating confirmation of the internal validity of the models.

Standard clinical or instrumented measures showed moderate discriminative ability with an equal AUC of 0.68 (95%CI [0.60–0.76], *p* = 0.97). Similar results were obtained for both models with either standard clinical or instrumented measures of TUG: AUC of 0.68, 95%CI [0.60–0.77] and AUC of 0.65, 95%CI [0.56–0.73], respectively, *p* = 0.26. 

The sensitivity analyses including the combined models of standard and instrumented features showed that no significant differences could be found between the standard clinical, instrumented or combined models (*p*-values all > 0.05), indicating equal ability to discriminate VHFS from HFS.

## 4. Discussion

This study aimed to compare the discriminative ability of standard clinical with instrumented measures of physical performance assessments in distinguishing between HFS and VHFS in a relatively healthy population of community-dwelling adults aged 61–70 years. The 30CST number of repetitions and TUG duration (recorded with stopwatch as well as by the smartphone) showed moderate discriminative ability. These two types of measurement showed similar performances in the univariable logistic regressions. The results suggest that identification of minor differences in functional status is possible in this relatively healthy population, either by standard clinical or instrumented measures recorded through a smartphone. Physical performance assessments instrumented by means of a smartphone allow us to collect a number of additional features beyond the number of repetitions (30CST) or total duration (TUG). These features could have the potential to add more detailed information on the participants’ functional status. 

For the 30CST assessment, three of the 30CST instrumented features were entered as input to fit the final model: “mean Duration Sit-to-Stand”, “SD Duration Sit-to-Stand”, and “mean Stand-to-Sit G RMS ML”. The 30CST, by definition, is a measure of lower limbs strength and endurance. The time needed to stand up from a sitting position represents the dynamic balance and can be considered as an index of the power generated from muscles to stand up against gravity. The shorter the duration, the higher the strength. The standard deviation (SD) of the duration is a measure of variability; the higher the SD, the higher the difference between the duration of this task among the repetitions. Indeed, high SD of the standing duration could be related to fatigue and weakness. The Stand-to-Sit G RMS in ML direction is a measure of the intensity of the forward trunk rotation while sitting. The sitting phase requires dynamic balance and lower limbs strength to control the lowering of the body to the seated position. A more intense trunk rotation during the Stand-to-Sit phase could be related to less muscle strength, as demonstrated in a recent study for the Sit-to-Stand phase [35].

The final model of the TUG included two instrumented features: “Walk duration” and “Turn-to-Sit Turning maximum velocity”. The duration of the straight walk is a predictor of health status in old age, and as such gait speed is commonly recorded to assess individuals’ functional abilities [36]. Difficulty in turning, i.e., slower turning velocity, has been associated with mild cognitive impairment in old age [37]. The turn before sitting differs from the 180Turn as it involves cognition, motor planning and visual capacities in preparation for sitting [37].

The DeLong test between the standard clinical and instrumented assessments did not result in significant differences between the types of assessments, suggesting that these two types of measurement have a similar discriminative ability. Yet, in contrast to the standard clinical measures, the instrumented features allow to objectively measure the participants’ capacities while performing specific (sub-) tasks, such as walking, turning, or sitting. Furthermore, the discriminative ability slightly increased, albeit not significantly, when the standard clinical and instrumented measures were combined, suggesting that the two types of assessment have small additional value in our target group. These results are in agreement with a recent study in which was demonstrate that standard clinical and instrumented measures of physical performance are associated with similar effect size to age-related changes in physical performance [38]. 

This study does have some limitations to consider. First, we included a rather homogeneous population, characterized by a highly skewed distribution of relatively high LLFDI scores, which may have led to a decrease in the discriminative ability of the models. Yet, even in this homogeneous and healthy population, we found discriminative value of both types of assessments for as well 30CST as TUG. The second limitation was our dichotomization based on the median value of the LLFDI scores, in absence of a validated cut-off for discriminating between different LLFDI levels. A valid cut-off score can be helpful to identify people at risk of developing functional decline. This aspect might be the subject of future studies. Despite these limitations, instrumented 30CST and TUG features proved to be comparable to the standard clinical measures, with moderate discriminative ability, in detecting even small differences of LLFDI in this homogeneous population of highly functioning individuals. It is reasonable to assume that the detection of differences in the functional status would also be possible in less fit and more heterogeneous population of older adults, yet this needs to be confirmed in future studies. For future perspectives, the potential of instrumented assessments may be preferred over standard clinical assessments for example in the context of self-management or Active Assisted Living Programmes. Therefore, we recommend further investigation of the sensitivity to changes over time of instrumented features, as well as of their correlations with measures of functional status and health obtained by other systems for monitoring activities of daily living, such as daily life gait speed.

## 5. Conclusions

In a relatively healthy population of adults aged 61–70 years, standard clinical and instrumented measures recorded through a smartphone can distinguish between HFS and VHFS, albeit with moderate discriminative ability.

## Figures and Tables

**Table 1:** Instrumented features extracted from the 30-s Chair Stand (30CST) test.

Feature	Sensor	(Sub)Phases	Description
Repetitions [number]	Accelerometer/ Gyroscope	Total	Total number of repetitions
SD Duration	Accelerometer/ Gyroscope	Sit-to-Stand, Stand-to-Sit subphases	Standard deviation of the duration of each subphase of the 30CST
Duration [s]	Accelerometer/ Gyroscope	Sit-to-Stand, Stand-to-Sit subphases	Duration of each subphase of the 30CST
NJS AP ML V [m]	Accelerometer	Sit-to-Stand, Stand-to-Sit subphases	Time-normalized Jerk Score of the acceleration: $NJS=\sqrt{\frac{T^{5}}{2}\int_{Tstart}^{Tend}{\left(\dot{a}\right)}^{2}dt}$ where *T* is the duration (*Tend-Tstart*) of the considered submovement and *a* is the acceleration measured in m/s^2^.
RMS AP, ML, V [m/s^2^], [°/s]	Accelerometer, Gyroscope	Sit-to-Stand, Stand-to-Sit subphases	Root Mean Square of the signal, *s*, during the considered submovement (hence a measure of dispersion): $RMS=\sqrt{\frac{1}{N}\overset{N}{\underset{i=1}{\sum }}{\left(s_{i}-m\right)}^{2}}$ where *N* is the total number of points of the signal s, and *m* is the mean value:

ACRONYMS: AP: Antero-Posterior; ML: Medio-Lateral; V: Vertical

**Table 2:** Instrumented features extracted from the Timed Up and Go (TUG) test.

Feature	Sensor	(Sub)Phases	Description
Duration [s]	Accelerometer/ Gyroscope	Total, Sit-to-Walk, Walk, 180Turn, Turn-to-Sit	Total duration and duration of each subphase of the TUG
Number of Steps	Accelerometer/ Gyroscope	180Turn, Walk	Number of steps during each subphase of the TUG
RMS AP, ML, V [m/s^2^]	Accelerometer	Sit-to-Walk, Walk, Turn-to-Sit	Root Mean Square of the signal, *s*, during the considered subphase (hence a measure of dispersion): $RMS=\sqrt{\frac{1}{N}{\sum_{i=1}^{N}(s}_{i}-m{)}^{2}}$ where *N* is the total number of points of the signal *s*, and *m* is the mean value: *mean(s)*
NJS AP, ML, V [m]	Accelerometer	Sit-to-Walk, Turn-to-Sit	Time-Normalized Jerk Score of the acceleration: $NJS=\sqrt{\frac{T^{5}}{2}\int_{Tstart}^{Tend}{\left(\dot{a}\right)}^{2}dt}$ where *T* is the turn duration (*Tend-Tstart*) of the considered subphase, *a* is the acceleration measured in m/s^2^.
NJS V [-]	Gyroscope	180Turn, Turn-to-Sit Turning	Normalized angular Jerk Score: $NJS=\sqrt{\frac{T^{5}}{2TA^{2}}\int_{Tstart}^{Tend}{\left(\overset{..}{\omega }\right)}^{2}dt}$; where *T* is the turn duration (*Tend-Tstart*) of the considered component, *ɷ* is the angular velocity °/s, and *TA* is the Turning Angle in °. $TA=\int_{Tstart}^{Tend}\omega dt$
Mean Velocity [°/s]	Gyroscope	180Turn, Turn-to-Sit Turning	Mean Velocity, as the mean value of the angular velocity along the vertical axis during the turn: $Mean\;Velocity=\frac{1}{NE-NS}\sum_{NE}^{i=NS}\omega (i)$ where ω is the angular velocity in °/s; *NE* and *NS* are the index of the end and the index of the beginning of the turn, respectively.
Maximum Velocity [°/s]	Gyroscope	180Turn, Turn-to-Sit Turning	Maximum Velocity as the maximum value of the angular velocity along the vertical axis during the turn: $Maximum\;Velocity=\max{\left(\omega \right)}_{NS}^{NE}$ where ω is the angular velocity in °/s; *NE* and NS are the index of the end and the index of the beginning of the turn, respectively.

ACRONYMS: AP: Antero-Posterior; ML: Medio-Lateral; V: Vertical

**Table 3:** Description of the population stratified by High Functional Status (HFS) and Very High Functional Status (VHFS).

	Total Population N = 160	HFS N = 78	VHFS N = 82
Gender, Female	87 (54.38%)	52 (66.67%)	35 (42.68%)
Age, years	66.29 (2.40)	66.13 (2.44)	66.45 (2.37)
Height, cm	170.94 (9.35)	169.32 (9.86)	172.49 (8.63)
Weight, kg	79.49 (15.61)	79.97 (16.35)	79.04 (14.95)
Handgrip strength, kg	34.41 (11.19)	31.06 (10.75)	37.61 (10.71)
Gait speed, m/s	2.05 (0.46)	1.82 (0.41)	2.27 (0.40)
30CST, number of repetitions	13.41 (3.29)	12.36 (3.13)	14.40 (3.14)
TUG duration, s	8.70 (1.60)	9.25 (1.85)	8.17 (1.10)
PA >=3	144 (90%)	71 (91.03%)	73 (89.02%)
Falls, number >=2	23 (14.38%)	15 (19.23%)	8 (9.76%)
MoCA, points	27.08 (1.85)	27.06 (1.89)	27.09 (1.83)
Medications, number >=4	44 (27.50%)	29 (37.18%)	15 (18.29%)
LLFDI, points, median [range]	72.31 [44.33 100]	65.57 [44.33 71.33]	79.35 [72.31 100]

Values are presented as mean (SD) or number (%) unless otherwise indicated. ACRONYMS: 30CST: 30-s Chair Stand test; HFS: High Functional Status; LLFDI: Late-Life Function and Disability Instrument; MoCA: Montreal Cognitive Assessment; PA: declared physical activity level; TUG: Timed Up and Go test; VHFS: Very High Functional Status.

**Table 4:** Sensitivity analysis.

		AUC	95% CI	*p*-Value of the DeLong Test	
30CST	Standard clinical	0.68	[0.60–0.76]	Standard clinical—Instrumented	0.97
	Instrumented	0.68	[0.60–0.76]	Instrumented—Combined	0.74
	Combined	0.69	[0.61–0.77]	Standard clinical—Combined	0.48
TUG	Standard clinical	0.68	[0.60–0.77]	Standard clinical—Instrumented	0.26
	Instrumented	0.65	[0.56–0.73]	Instrumented—Combined	0.94
	Combined	0.69	[0.60–0.77]	Standard clinical—Combined	0.12

## Supplementary Materials

The following are available online at http://www.mdpi.com/1424-8220/19/3/449/s1, Table S1: Collinearity analysis of the 30CST instrumented physical performance measures; Table S2: Univariable and multivariable analysis of the 30CST instrumented physical performance measures; Table S3: Collinearity analysis of the TUG instrumented physical performance measures; Table S4: Univariable and multivariable analysis of the TUG instrumented physical performance measures; Table S5: Bootstrapping validation of the 30CST and TUG models.
