# Supplementary material for: Comparison of Standard Clinical and Instrumented Physical Performance Tests in Discriminating Functional Status of High-Functioning People Aged 61–70 Years Old
Source: Sensors (Basel). 2019 Jan 22;19(3):449. doi: 10.3390/s19030449 (PMC6387343; doi:10.3390/s19030449)
Supplement: Supplementary file 1 [file sensors-19-00449-s001.pdf]

sensors

Article

Comparison of Standard Clinical and Instrumented Physical Performance Tests in Discriminating Functional Status of High-Functioning People Aged 61–70 Years Old

Alice Coni [1], Jeanine M. Van Ancum [2], Ronny Bergquist [3], A. Stefanie Mikolaizak [4], Sabato Mellone [1,5], Lorenzo Chiari [1,5], Andrea B. Maier [2,6], Mirjam Pijnappels [2,*]

1 Department of Electrical, Electronic and Information Engineering "Guglielmo Marconi" (DEI), University of Bologna, 40136 Bologna, Italy; alice.coni2@unibo.it (A.C.); sabato.mellone@unibo.it (S.M.); lorenzo.chiari@unibo.it (L.C.)
2 Department of Human Movement Sciences, @AgeAmsterdam, Faculty of Behavioural and Movement Sciences, Vrije Universiteit Amsterdam, Amsterdam Movement Sciences, 1081 BT Amsterdam, The Netherlands; j.m.van.ancum@vu.nl (J.M.V.A.); Andrea.Maier@mh.org.au (A.B.M.)
3 Department of Neuromedicine and Movement Science, Norwegian University of Science and Technology, 7491, Trondheim, Norway; ronny.bergquist@ntnu.no
4 Department of Clinical Gerontology, Robert Bosch Medical Foundation, 70376 Stuttgart, Germany; Stefanie.Mikolaizak@rbk.de
5 Health Sciences and Technologies—Interdepartmental Center for Industrial Research (HST-ICIR), University of Bologna, 40126 Bologna, Italy
6 Department of Medicine and Aged Care, @AgeMelbourne, University of Melbourne, Royal Melbourne Hospital, 3050 Melbourne, Australia
* Correspondence: m.pijnappels@vu.nl

Received: 30 November 2018; Accepted: 19 January 2019; Published: 22 January 2019

Table S1. Collinearity analysis of the 30CST instrumented physical performance measures.

	First step		Last step	
	**VIF**	**detection**	**VIF**	**detection**
Mean Sit-to-Stand RMS A AP	5.79	0	3.30	0
Mean Sit-to-Stand RMS A ML	6.01	0	2.91	0
Mean Sit-to-Stand RMS A V	4.68	0	2.45	0
Mean Sit-to-Stand NJS A AP	20.26	1	6.06	0
Mean Sit-to-Stand NJS A ML	18.54	1	-	-
Mean Sit-to-Stand NJS A V	17.02	1	-	-
Mean Sit-to-Stand RMS G AP	3.28	0	2.85	0
Mean Sit-to-Stand RMS G ML	4.27	0	3.88	0
Mean Stand-to-Sit RMS A AP	7.51	0	2.78	0
Mean Stand-to-Sit RMS A ML	7.31	0	3.16	0
Mean Stand-to-Sit RMS A V	4.14	0	2.09	0
Mean Stand-to-Sit NJS A AP	29.03	1	2.44	0
Mean Stand-to-Sit NJS A ML	27.97	1	-	-
Mean Stand-to-Sit NJS A V	19.68	1	-	-
Mean Stand-to-Sit RMS G AP	4.38	0	3.73	0
Mean Stand-to-Sit RMS G ML	5.34	0	3.86	0
Mean Duration Sit-to-Stand	11.18	1	4.84	0
SD Duration Sit-to-Stand	5.70	0	5.27	0
Mean Duration Stand-to-Sit	17.27	1	-	-
SD Duration Stand-to-Sit	4.26	0	3.77	0
Instrumented number of repetitions	24.75	1	-	-

ACRONYMS: A: Accelerometer; AP: Antero-Posterior; G=Gyroscope; ML: Medio-Lateral; RMS: Root Mean Square; SD: Standard Deviation; NJS: Normalized Jerk Score; V: Vertical; VIF: Variance Inflation Factor

Table S2. Univariable and multivariable analysis of the 30CST instrumented physical performance measures.

	Univariable logistic regression			Stepwise backward multivariable logistic regression		
	**OR**	**95% CI**	**p-value**	**OR**	**95% CI**	**p-value**
Mean Sit-to-Stand RMS A AP	1.04	[0.76-1.41]	0.820			
Mean Sit-to-Stand RMS A ML	1.22	[0.89-1.68]	0.224			
Mean Sit-to-Stand RMS A V	1.12	[0.82-1.53]	0.473			
Mean Sit-to-Stand NJS A AP[1]	0.80	[0.58-1.09]	0.157			
Mean Sit-to-Stand RMS G AP	1.01	[0.74-1.38]	0.928			
Mean Sit-to-Stand RMS G ML	1.14	[0.83-1.56]	0.413			
Mean Stand-to-Sit RMS A AP	1.03	[0.76-1.40]	0.852			
Mean Stand-to-Sit RMS A ML	1.14	[0.83-1.56]	0.415			
Mean Stand-to-Sit RMS A V	1.12	[0.82-1.53]	0.487			
Mean Stand-to-Sit NJS A AP[1]	0.67	[0.49-0.94]	**0.019**			
Mean Stand-to-Sit RMS G AP	0.90	[0.66-1.23]	0.503			
Mean Stand-to-Sit RMS G ML	0.78	[0.57-1.08]	**0.131**	0.71	[0.49 0.98]	**0.045**
Mean Duration Sit-to-Stand	0.59	[0.41-0.84]	**0.004**	0.69	[0.48 0.98]	**0.041**
SD Duration Sit-to-Stand	0.65	[0.47-0.92]	**0.014**	0.62	[0.41 0.89]	**0.014**
SD Duration Stand-to-Sit	0.82	[0.60-1.13]	0.226			

Bolded p-values indicate statistically significant univariable and multivariable discriminative ability (≤ 0.15 and ≤ 0.05 respectively).

ACRONYMS: A: accelerometer; AP: Antero-Posterior; G: gyroscope; NJS: Normalized Jerk Score; ML: Medio-Lateral; RMS: Root Mean Square; SD: Standard Deviation; V: Vertical. [1]log transformed feature

Table S3. Collinearity analysis of the TUG instrumented physical performance measures.

	**First step**		**Last step**	
	**VIF**	**detection**	**VIF**	**detection**
Sit-to-Walk Duration	11.44	1	6.20	0
180Turn Duration	12.11	1	6.90	0
Turn-to-Sit Turning Duration	9.43	0	9.35	0
Turn-to-Sit Duration	37.85	1	9.36	0
Walk Duration	79.06	1	7.67	0
Sit-to-Walk RMS A AP	2.91	0	2.90	0
Sit-to-Walk RMS A ML	2.91	0	2.83	0
Sit-to-Walk RMS A V	18.24	1	2.31	0
Sit-to-Walk NJS A AP	10.17	1	9.97	0
Sit-to-Walk NJS A ML	8.11	0	8.02	0
Sit-to-Walk NJS A V	7.58	0	6.75	0
Turn-to-Sit RMS A AP	2.77	0	2.36	0
Turn-to-Sit RMS A ML	4.54	0	2.98	0
Turn-to-Sit RMS A V	24.83	1	-	-
Turn-to-Sit NJS A AP	10.57	1	9.08	0
Turn-to-Sit NJS A ML	12.85	1	-	-
Turn-to-Sit NJS A V	8.31	0	6.43	0
180Turn Mean Velocity	8.65	0	8.23	0
Turn-to-Sit Turning Mean Velocity	9.82	0	9.70	0
180Turn Maximum Velocity	3.74	0	3.41	0
Turn-to-Sit Turning Maximum Velocity	6.01	0	5.97	0
180Turn NJS G V	3.58	0	3.39	0
Turn-to-Sit Turning NJS G V	4.39	0	4.34	0
Walk RMS A AP	3.66	0	3.11	0
Walk RMS A ML	2.71	0	2.52	0
Walk RMS A V	18.69	1	-	-
180Turn Number of Steps	2.46	0	2.41	0
Walk Number of Steps	6.88	0	6.43	0
Instrumented TUG total duration	132.41	1	-	-

ACRONYMS: A: Accelerometer; AP: Antero-Posterior; G=Gyroscope; ML: Medio-Lateral; NJS: Normalized Jerk Score; RMS: Root Mean Square; SD: Standard Deviation; V: Vertical; VIF: Variance Inflation Factor

Table S4. Univariable and multivariable analysis of the TUG instrumented physical performance measures.

	**Univariable**			**Stepwise backward multivariable logistic regression**		
	**OR**	**95% CI**	**p-value**	**OR**	**95% CI**	**p-value**
Sit-to-Walk Duration	0.96	[0.70-1.31]	0.786			
180Turn Duration	0.80	[0.58-1.11]	0.185			
Turn-to-Sit Turning Duration	0.62	[0.44-0.88]	**0.008**			
Turn-to-Sit Duration	0.70	[0.50-0.97]	**0.032**			
Walk Duration	0.54	[0.36-0.79]	**0.002**	0.59	[0.38-0.86]	**0.010**
Sit-to-Walk RMS A AP	1.20	[0.87-1.65]	0.258			
Sit-to-Walk RMS A ML	1.04	[0.76-1.43]	0.787			
Sit-to-Walk RMS A V	1.89	[0.69-5.18]	0.213			
Sit-to-Walk NJS A AP[1]	1.18	[0.86-1.63]	0.303			
Sit-to-Walk NJS A ML[1]	1.16	[0.84-1.60]	0.364			
Sit-to-Walk NJS A V[1]	1.28	[0.90-1.82]	0.173			
Turn-to-Sit RMS A AP	0.96	[0.71-1.31]	0.805			
Turn-to-Sit RMS A ML	1.25	[0.91-1.72]	0.164			
Turn-to-Sit NJS A AP[1]	0.94	[0.69-1.28]	0.703			
Turn-to-Sit NJS A V[1]	0.82	[0.60-1.13]	0.223			
180Turn Mean Velocity	1.18	[0.86-1.62]	0.301			
Turn-to-Sit Turning Mean Velocity	1.60	[1.14-2.25]	**0.007**			
180Turn Maximum Velocity	1.38	[1.00-1.91]	**0.051**			
Turn-to-Sit Turning Maximum Velocity	1.66	[1.17-2.35]	**0.004**	1.50	[1.05-2.18]	**0.031**
180Turn NJS G V[1]	0.87	[0.63-1.19]	0.386			
Turn-to-Sit Turning NJS G V[1]	0.76	[0.55-1.06]	**0.104**			
Walk RMS A AP	1.35	[0.95-1.92]	**0.098**			
Walk RMS A ML	1.26	[0.92-1.74]	0.155			
180Turn Number of Steps	0.95	[0.70-1.31]	0.764			
Walk Number of Steps	0.58	[0.40-0.85]	**0.005**			

Bolded p-values indicate statistically significant univariable and multivariable discriminative ability (≤ 0.15 and ≤ 0.05 respectively).

ACRONYMS: A: accelerometer; AP: Antero-Posterior; G: gyroscope; ML: Medio-Lateral; NJS: Normalized Angular Jerk Score; RMS: Root mean square; V: Vertical; [1]log transformed feature.

Table S5. Bootstrapping validation of the 30CST and TUG models.

	**30CST**		**TUG**	
	**AUC original**	**AUC corrected**	**AUC original**	**AUC corrected**
Standard clinical	0.682	0.684	0.684	0.685
Instrumented	0.680	0.654	0.650	0.627
Combined	0.661	0.630	0.684	0.670
